# A meta-analysis: postoperative prophylactic antibiotics compared with non-antibiotics for pediatric hypospadias repair

**DOI:** 10.3389/fmed.2026.1718769

**Published:** 2026-01-29

**Authors:** Fengming Ji, Hongjing Jiang, Yihong Li, Yu Hang, Jinrong Li, Chengchuang Wu, Bing Yan, Chenghao Zhanghuang

**Affiliations:** 1 Urology Surgery Department of the Children’s Hospital of Kunminng Medical University, Kunming Chlidren’s Hospital, Key Laboratory of Children’s Major Disease Research, Kunming, Yunnan, China; 2 Dermatology Department of the Children’s Hospital of Kunminng Medical University, Kunming Chlidren’s Hospital, Key Laboratory of Children’s Major Disease Research, Kunming, Yunnan, China; 3 Anesthesiology Department of the Children’s Hospital of Kunminng Medical University, Kunming Chlidren’s Hospital, Key Laboratory of Children’s Major Disease Research, Kunming, Yunnan, China

**Keywords:** antibiotics, complications, hypospadias, meta-analysis, urinary tract infection

## Abstract

**Objective:**

This study evaluated the impact of postoperative prophylactic antibiotics (PA) use on complications after hypospadias surgery, to guide clinical diagnosis and treatment, as well as promote the rational application of PA.

**Method:**

Through computer searches of PubMed, EMbase and Cochrane Library, randomized controlled trial (RCT) or non-randomized controlled trial (NRCT) on the postoperative PA for hypospadias from the year 2000 to the present were included. The included studies divided the subjects into antibiotics and non-antibiotics groups based on whether PA was used postoperatively. Data analysis was performed using RevMan 5.4 and STATA 18.0 software, determining the odds ratio (OR) and 95% confidence interval (CI) through fixed-effect or random-effect models.

**Results:**

A total of seven studies were included in the research, comprising five RCT and two NRCT, involving 862 participants. Among these, 462 participants received antibiotics, with 53 experiencing complications. Two hundred and fifty-nine participants did not receive antibiotics, with 31 experiencing complications. The results of the meta-analysis indicated that there was a significant difference in the incidence of urinary tract infections (UTI) between the antibiotics group and the non-antibiotics group (*p* = 0.004), while there were no differences in the rates of overall complications (OC) (*p* = 0.61), fistula (*p* = 0.96), meatal stenosis (MS) (*p* = 0.40), symptomatic UTI (sUTI) (*p* = 0.55), SSI (*p* = 0.47), dehiscence (*p* = 0.27), and diverticulum (*p* = 0.98).

**Conclusion:**

This meta-analysis demonstrated that postoperative PA significantly reduced the incidence of UTI following pediatric hypospadias repair. However, no significant benefits were observed for OC, fistula, MS, sUTI, SSI, dehiscence, or diverticulum. The clinical relevance of this UTI reduction may be limited as it primarily reflects decreased asymptomatic bacteriuria rather than symptomatic infections. These findings suggest that routine postoperative PA use should be reconsidered, and individualized, risk-stratified approaches are needed.

## 1 Introduction

Hypospadias, a common congenital anomaly in male children, has an incidence rate ranging from 0.6 to 464 per 10,000 births (1, 2). Surgery is the exclusive therapeutic approach to restore normal micturition, sexual function, and to attain satisfactory plastic outcomes in hypospadias (3). Over the decades, more than 300 surgical techniques have been developed. Regardless of the chosen method, the use of indwelling catheters postoperatively is a common practice among surgeons. The most common complications after urethroplasty include urinary tract infection (UTI) and surgical site infections (SSI), each of which occurs in 3–7% of cases (4). Perioperative prophylactic antibiotics aims to reduce the risk of UTI and SSI (5, 6). At present, there are no definitive guidelines or standardized protocols for antimicrobial use following urethroplasty or urethral stent placement (7). The absence of guidelines means that physicians often rely on personal experience when prescribing medications, escalating the risk of antibiotic resistance in our patient cohort (8, 9).

In light of this, we conducted a meta-analysis and systematic literature review to investigate the correlation between postoperative PA use and the success of hypospadias surgery. Our goal was to determine the requirement and establish protocols for postoperative PA use following hypospadias surgery, to inform the creation of subsequent infection prevention strategies.

## 2 Literature and methods

### 2.1 Search strategy

The databases searched included PubMed, EMbase and Cochrane Library. The search covered the period from January 1, 2000, to the present, with language restrictions set for English. The English search terms were “antibiotics,” “prophylactic antibiotics,” and “hypospadias.” Boolean logic operators were used to formulate the search queries. The English search query was ((“antibiotics” or “prophylactic antibiotics”) and “hypospadias”).

### 2.2 Inclusion and exclusion criteria

#### 2.2.1 Inclusion criteria

(1) Participants: Pediatric patients (aged 0–18 years) undergoing surgical repair for hypospadias, regardless of the severity of the condition, with no known allergies to antibiotics or contraindications for antibiotic use. (2) Interventions: The experimental group received prophylactic antibiotics (specific classes such as cephalosporins, penicillins, or macrolides) administered preoperatively, with dosage and duration consistent with standard clinical guidelines for surgical prophylaxis. (3) Comparator: The control group received no antibiotics (placebo or standard care without antibiotics) prior to hypospadias repair. (4) Outcomes: At least one of the following outcomes was reported: overall complications (OC), fistula, meatal stenosis (MS), UTI, symptomatic UTI (sUTI), dehiscence and diverticulum. (5) Study design: Randomized controlled trials (RCT) and non-randomized controlled trials (NRCT) with a clear comparison between antibiotic use and non-use, with a minimum follow-up duration of 30 days post-surgery.

#### 2.2.2 Exclusion criteria

(1) Non-English literature or studies not published in peer-reviewed journals. (2) Studies involving adult patients (aged 19 years and older) or those with other congenital anomalies or conditions that may affect UTI rates or surgical outcomes. (3) Studies lacking full-text access or with incomplete data on outcomes of interest. (4) Studies without a clear comparison between antibiotic use and non-use or those that do not meet the minimum follow-up duration of 30 days post-surgery. (5) Animal studies, *in vitro* studies, reviews, meta-analyses, case reports, or letters to the editor.

### 2.3 Data extraction

Two investigators independently screened the literature and extracted data; discrepancies were resolved through consultation with a third investigator. The following variables were extracted: author and year of publication, study design, study period, subject age, antibiotic administration routes, preoperative antibiotic prophylaxis, hypospadias classification, and catheter indwelling duration.

### 2.4 Assessment of risk bias of included studies

Risk of bias assessment was independently performed by two reviewers, with discrepancies resolved by consensus or adjudication by a third reviewer. RCTs were evaluated using the Cochrane Collaboration’s Risk of Bias tool, while non-randomized studies were assessed with the Newcastle–Ottawa Scale.

### 2.5 Statistical analysis

All statistical analyses were conducted using RevMan 5.4 software and STATA 18.0. Heterogeneity among the results of the included studies was analyzed using the 𝜒^2^ test, and the *I*^2^ was used to quantitatively assess the degree of heterogeneity. If there is no statistical heterogeneity among the study results (*I*^2^ < 50%), a fixed-effects model will be used for the meta-analysis. If there is statistical heterogeneity among the study results (*I*^2^ > 50%), a random-effects model will be used for the meta-analysis. All effect sizes are presented with 95% confidence intervals (95% CI), and a *p*-value of less than 0.05 indicates statistical significance.

## 3 Results

### 3.1 Results of literature search

**Figure 1:**
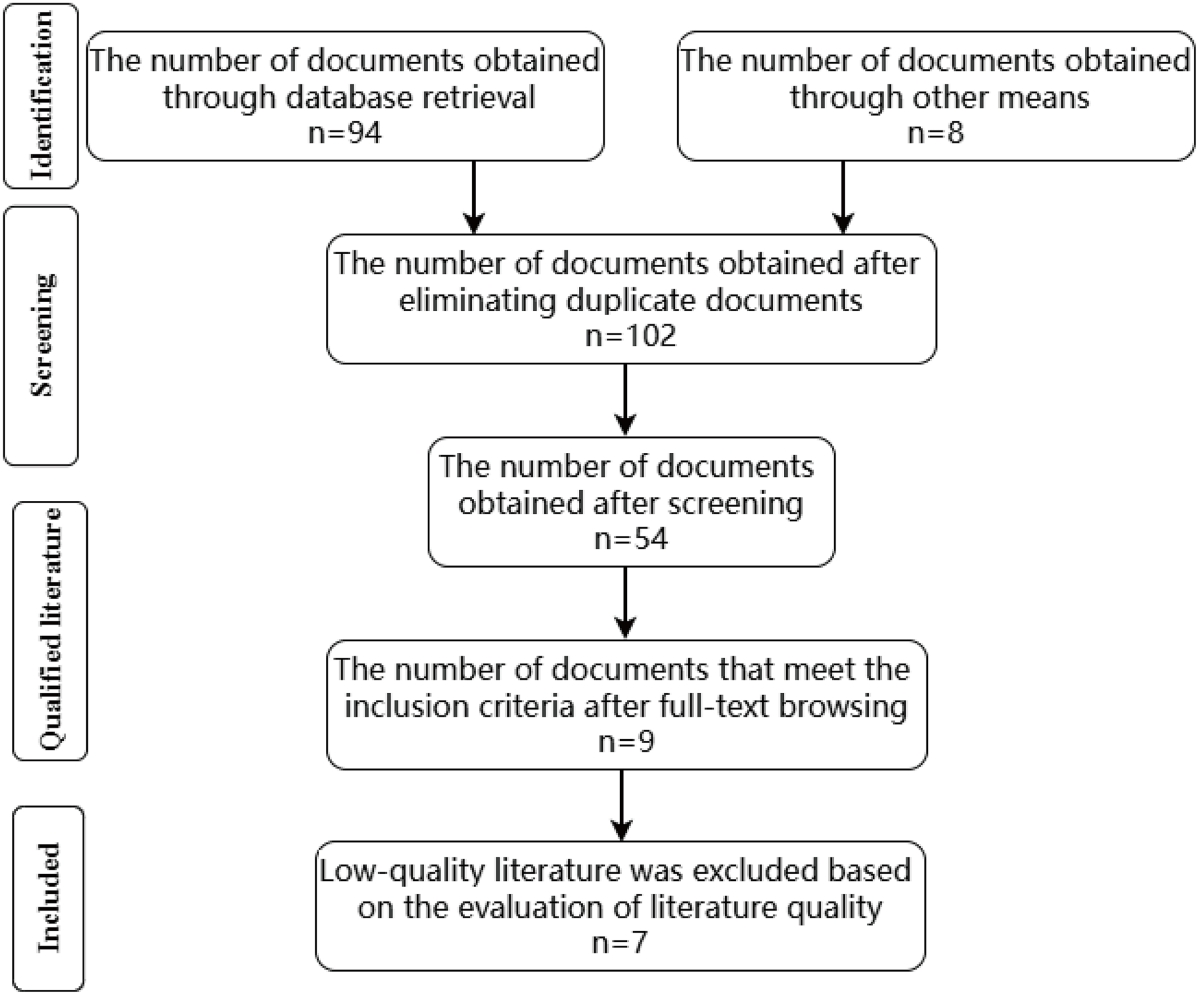
Literature screening process and results.

A total of 94 articles were identified through the database search, and an additional eight articles were obtained from the references of retrieved studies. After removing duplicates, 102 articles remained. Following initial screening, case reports, reviews, systematic reviews, animal studies, conference abstracts, and other irrelevant study types were excluded, leaving 54 articles for further assessment. After full-text review based on the inclusion and exclusion criteria, nine articles were selected. Two studies were subsequently excluded due to low methodological quality. Ultimately, seven articles were included in this meta-analysis. (The literature screening process is shown in Figure 1, basic characteristics of included studies were shown in Table 1).

**Table 1:** Basic characteristics of included studies.

References	Type of study	Study period	Age	RA	Type of hypospadias	DIC (d)
Glaser et al. (9)	RCT	/	11 months–6.5 years	Oral	G:C:P:PS 6:27:16:3 vs. 3: 27:17:2	5–14
Basin et al. (10)	RCT	2013/6–2017/5	6 months–2 years	Oral	G:M:D 6:2:27 vs. 4:2:26	6–8
Zhang et al. (11)	RCT	2014/3–2018/6	8 months–11 months	Oral	G:C:P:PS 2:19:5:19 vs. 2:19:5:22	6–8
Cahill et al. (4)	NRCT	2009/9–2012/1	0.6 years–14 years	Oral	G:C:M:D 10:47:12:9 vs.16:39:8:8	15
He et al. (12)	RCT	2015/1–2017/2	0.8 ± 0.7 years	Oral	G:C:D 7:14:27 vs. 4:7:13	5–8
Pogorelić et al. (13)	NRCT	2011/1–2017/2	14.55 ± 11.10 months	Oral	/	5–8
Esposito et al. (14)	RCT	2020/8–2022/1	6 months–12 years	Injection + oral	/	5–10

RA, route of administration; DIC, duration of indwelling catheter; RCT, randomized controlled trial; NRCT, non-randomized controlled trial; G, glanular; C, coronal; P, penile; M, midshaft; PS, penoscrotal; D, distal shaft.

### 3.2 Results of meta-analysis

#### 3.2.1 OC

**Figure 2:**
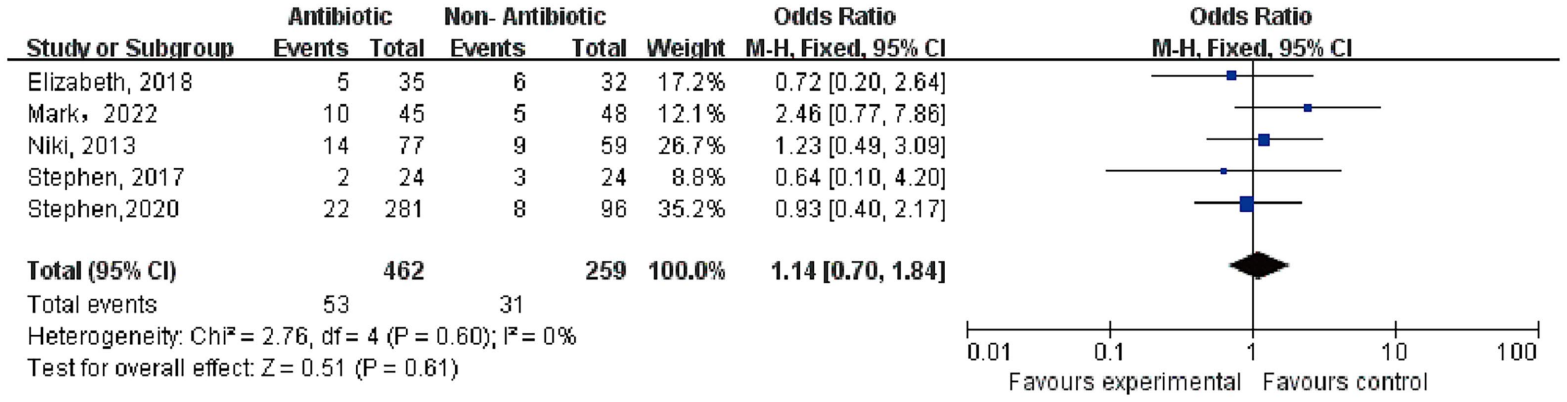
Meta-analysis results of overall complications.

A total of five studies reported on OC. In the antibiotic group, there were 462 cases with 53 complications, while in the non-antibiotic group, there were 259 cases with 31 complications. The heterogeneity test showed *p* = 0.60, *I*^2^ = 0%, indicating no significant heterogeneity. A fixed-effect model was used, and the result indicated OR = 1.14, 95% CI = 0.07–1.84, *p* = 0.61, suggesting no significant difference in the OC rates between the two groups. (The results of the meta-analysis of OC was shown in Figure 2).

#### 3.2.2 Other complications

**Figure 3:**
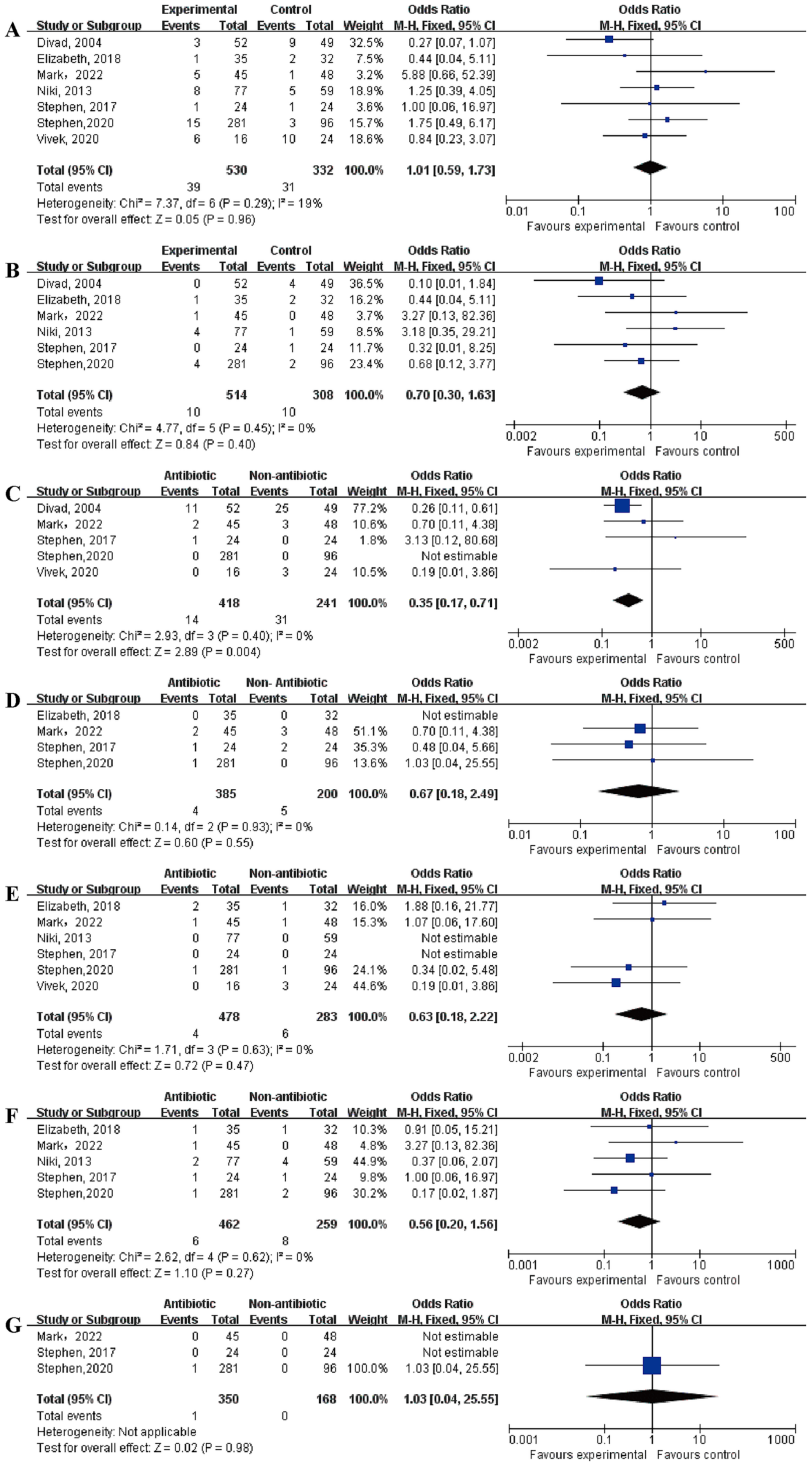
Meta-analysis results of other complications: **(A)** fistula; **(B)** meatal stenosis; **(C)** urinary tract infections; **(D)** symptomatic urinary tract infections; **(E)** surgical site infections; **(F)** dehiscence; **(G)** diverticulum.

According to the meta-analysis results, only UTI showed a statistically significant difference between the two groups. A total of five articles reported on UTI. In the antibiotic group, there were 418 cases with 14 complications, while in the non-antibiotic group, there were 241 cases with 31 complications. The heterogeneity test showed *p* = 0.40, *I*^2^ = 0%, and a fixed-effect model was used and the result indicated OR = 0.35, 95% CI = 0.17–0.71, *p* = 0.004. There were no significant differences in the incidence rates of fistula, MS, UTI, sUTI, SSI, dehiscence, and diverticulum. (The results of the meta-analysis were shown in Table 2 and Figure 3).

**Table 2:** The results of the meta-analysis.

Complications	Antibiotic		Non-antibiotic		Heterogeneity		Test for overall effect		
	Events	Total	Events	Total	*p*	*I*^2^ (%)	*Z*	*p*	OR (95% CI)
OC (4, 10–13)	53	462	31	259	0.60	0	0.51	0.61	1.14 (0.70–1.84)
Fistula (4, 9–14)	39	530	31	332	0.29	19	0.05	0.96	1.01 (0.59–1.73)
MS (4, 9–13)	10	514	10	308	0.45	0	0.84	0.40	0.70 (0.30–1.63)
UTI (9, 11–14)	14	418	31	241	0.40	0	2.89	0.004	0.35 (0.17–0.71)
sUTI (10–13)	4	385	5	200	0.93	0	0.60	0.55	0.67 (0.18–2.49)
SSI (4, 10–14)	4	478	6	283	0.63	0	0.72	0.47	0.63 (0.18–2.22)
Dehiscence (4, 10–13)	6	462	8	259	0.62	0	1.10	0.27	0.56 (0.20–1.56)
Diverticulum (11–13)	1	350	0	168	/	/	0.02	0.98	1.03 (0.04–25.55)

OR, odds ratio; CI, confidence interval; OC, overall complications; MS, meatal stenosis; UTI, urinary tract infections; sUTI, symptomatic urinary tract infections; SSI, surgical site infections.

#### 3.2.3 Publication bias analysis

**Figure 4:**
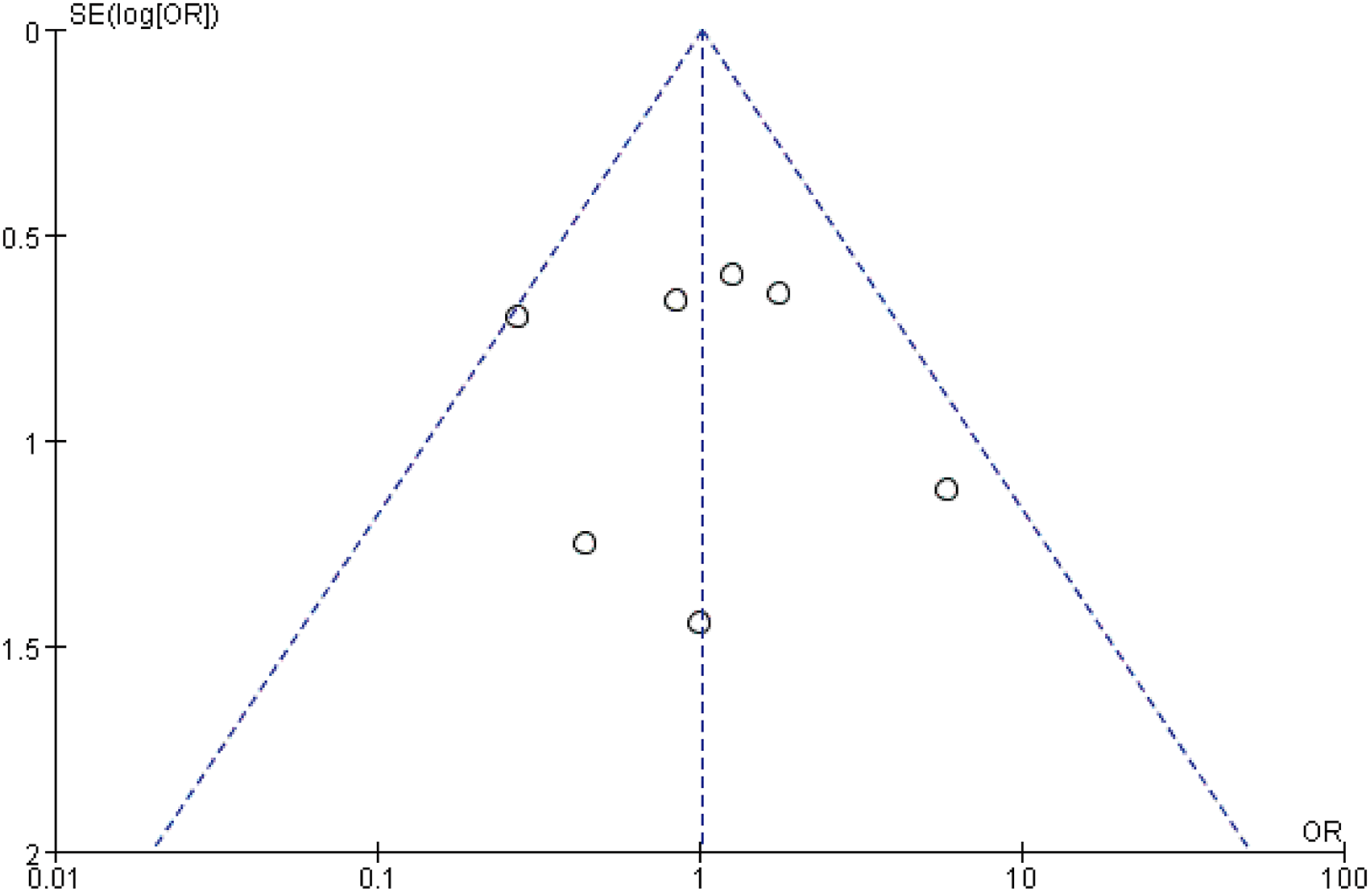
Funnel plot of publication bias.

Funnel plot analysis was conducted using STATA 18.0 software, and the funnel plot appeared to be symmetrical. (Publish bias funnel plot was shown in Figure 4). An Egger’s test was performed, which showed: *t* = 2.09, *p* = 1.015, indicating symmetry in the funnel plot. This suggests that there was no publication bias among the included studies.

## 4 Discussion

In the ongoing exploration of hypospadias treatment, despite continuous improvements in surgical techniques and increasingly advanced materials, the overall success rate of hypospadias surgery remains unsatisfactory (10). Particularly, the management of severe hypospadias continues to be a challenging frontier in pediatric urology that urgently requires breakthroughs (11). A major challenging issue of hypospadias is the high incidence of postoperative complications (12, 13). Our study results showed that postoperative PA only significantly reduced the incidence of UTI, but had no significant effect on the occurrence of OC, sUTI, fistula, or diverticula. This suggests that the benefit of PA is mainly focused on reducing the “quantity” of bacteriuria rather than altering its “quality.”

The statistically significant reduction in overall UTI contrasts sharply with the lack of effect on sUTI (*p* = 0.55). This divergence suggests that PA predominantly suppresses asymptomatic bacteriuria—a common colonization phenomenon in catheterized patients—without meaningfully impacting clinically consequential infections characterized by fever, dysuria, or systemic signs. The development of postoperative UTI after hypospadias repair is intimately linked to urethral manipulation, indwelling catheterization, and mucosal edema at the operative site (14). These insults disrupt the innate urethral barrier and facilitate bacterial colonization (15, 16). By suppressing or eradicating potential pathogens within the urethra, PA can effectively reduce the incidence of bacteriuria during the early postoperative period (17, 18). However, the use of PA in hypospadias surgery has always been a highly controversial topic. Many surgeons continue to administer antibiotics before and after the procedure to reduce the risk of infection, current evidence does not clearly support this practice. A retrospective study found that PA did not significantly lower the incidence of SSI among patients undergoing hypospadias repair (19). Another study based on the National Surgical Quality Improvement Program also showed that the use of a single PA was not significantly associated with postoperative complications (10).

Strategies for preventing SSI involve a multi-phase approach spanning the preoperative, intraoperative, and postoperative periods. A thorough preoperative evaluation and preparation should be conducted, including optimizing the patient’s nutritional status, managing underlying medical conditions, and performing appropriate skin disinfection (7). During surgery, strict aseptic techniques should be followed, operative time should be minimized as much as possible, and appropriate suture materials and techniques should be used (20). Postoperative care is equally important, involving regular inspection of the incision, timely management of exudate and signs of infection, and the use of antibiotics for prevention or treatment as needed (21).

Preventing and managing UTI following hypospadias surgery involves a comprehensive approach that goes beyond pharmacological interventions, integrating a spectrum of non-pharmacological preventative and therapeutic measures. Qin et al. (22) conducted an RCT study showing that expanding perineal cleaning can lower the incidence of UTI in comatose patients, female patients, and diabetic patients with short-term catheterization (≤10 days). The duration of catheter indwelling is a significant risk factor for UTI. Shortening the catheter indwelling time or timely replacing the catheter when necessary are effective measures to reduce the incidence of UTI (23, 24). Additionally, strictly evaluating the necessity of catheter placement and reducing non-essential catheterization events can also effectively prevent the occurrence of UTI (25, 26). Our meta-analysis showed that postoperative PA significantly reduced the incidence of UTI, yet it conferred no apparent benefit for other important complications such as fistula, MS, or dehiscence. Importantly, the observed reduction in UTI appears to reflect a decrease in asymptomatic bacteriuria rather than in sUTI, limiting its clinical relevance. When weighed against the modest advantage, the potential harms of antibiotic overuse, including gastrointestinal disturbance, allergic reactions and the emergence of resistant organisms, argue for a more refined, risk-stratified strategy (27–29).

The discovery of penicillin greatly revolutionized the treatment of infectious diseases, the subsequent excessive use of antibiotics has led to the emergence of antimicrobial resistance, now recognized as a significant global health challenge (30, 31). In the context of hypospadias surgery, where postoperative SSI and UTI are concerning complications that could be preventable, the regular postoperative PA is still a contentious issue. Accumulating evidence indicates that non-pharmacological measures may prevent UTI just as effectively without fostering resistance (28). Future investigations should therefore focus on developing individualized antibiotic protocols that account for patient-specific factors such as hypospadias severity, operative technique and duration of catheterization (32). Furthermore, large-scale multicenter RCT with standardization outcome definitions and prolonged follow-up are urgently required to provide robust evidence for clinical guidelines.

The limitations of this study include the small number of included articles and subjects. Additionally, simply dividing the subjects into antibiotic and non-antibiotic groups based on whether antibiotics were used seems overly simplistic. Conducting subgroup analyses based on the severity of hypospadias, surgical methods, and duration of catheter indwelling may yield more convincing and targeted results. Future studies still require more large-sample randomized controlled trials, in order to develop more personalized postoperative anti-infection plans for patients.

## 5 Conclusion

Based on this meta-analysis of seven studies involving 862 pediatric patients, postoperative PA after hypospadias repair significantly reduced the incidence of UTI but provided no demonstrable benefit for reducing sUTI, SSI, fistula, meatal stenosis, dehiscence, or diverticulum. The reduction in UTI primarily reflects decreased asymptomatic bacteriuria, which has limited clinical significance. Given the global challenge of antimicrobial resistance and the potential adverse effects of antibiotic overuse, our findings support a paradigm shift away from routine postoperative PA. Instead, we advocate for individualized decision-making that considers patient-specific risk factors, surgical technique, and duration of catheterization, while emphasizing non-pharmacological preventive measures such as perineal hygiene optimization and minimization of catheter dwell time. Large-scale, multicenter RCTs with standardized outcome definitions are warranted to establish evidence-based, personalized antibiotic protocols in pediatric hypospadias surgery.

## Data Availability

The original contributions presented in the study are included in the article/supplementary material, further inquiries can be directed to the corresponding author.
